# Multi-task genomic prediction using gated residual variable selection neural networks

**DOI:** 10.1186/s12859-025-06188-z

**Published:** 2025-07-07

**Authors:** Yuhua Fan, Patrik Waldmann

**Affiliations:** Research Unit of Mathematical Sciences, University of Oulu, P.O. Box 8000, Oulu, 90014 Finland

**Keywords:** Genomic selection, Gated residual neural networks, Variable selection, Deep learning

## Abstract

**Background:**

The recent development of high-throughput sequencing techniques provide massive data that can be used in genome-wide prediction (GWP). Although GWP is effective on its own, the incorporation of traditional polygenic pedigree information into GWP has been shown to further improve prediction accuracy. However, most of the methods developed in this field require that individuals with genomic information can be connected to the polygenic pedigree within a standard linear mixed model framework that involves calculation of computationally demanding matrix inverses of the combined pedigrees. The extension of this integrated approach to more flexible machine learning methods has been slow.

**Methods:**

This study aims to enhance genomic prediction by implementing gated residual variable selection neural networks (GRVSNN) for multi-task genomic prediction. By integrating low-rank information from pedigree-based relationship matrices with genomic markers, we seek to improve predictive accuracy and interpretability compared to conventional regression and deep learning (DL) models. The prediction properties of the GRVSNN model are evaluated on several real-world datasets, including loblolly pine, mouse and pig.

**Results:**

The experimental results demonstrate that the GRVSNN model outperforms traditional tabular genomic prediction models, including Bayesian regression methods and LassoNet. Using genomic and pedigree information, GRVSNN achieves a lower mean squared error (MSE), and higher Pearson (*r*) and distance (dCor) correlation between predicted and true phenotypic values in the test data. Moreover, GRVSNN selects fewer genetic markers and pedigree loadings which improves interpretability.

**Conclusion:**

The suggested GRVSNN framework provides a novel and computationally effective approach to improve genomic prediction accuracy by integrating information from traditional pedigrees with genomic data. The model’s ability to conduct multi-task predictions underscores its potential to enhance selection processes in agricultural species and predict multiple diseases in precision medicine.

## Background

Traditionally, quantitative genetics has been based on pedigree methods concentrated on the calculation of genetic relationships to predict polygenic breeding values. These methods rely on additive and dominance relationship matrices derived from known pedigrees in animal, plant and human studies [1]. The primary statistical framework for inference in such studies has been the linear mixed model, implemented through frequentist restricted maximum likelihood (REML) or Bayesian methods [2]. Recently, genome-wide prediction (GWP) has become a major part of many plant and animal breeding programs by taking advantage of massive genomic data to enhance phenotype selection [3, 4]. GWP also extends to human genetics, where it facilitates the prediction of disease risk scores [5].

With the advent of high-throughput sequencing, the integration of genomic markers, for example single nucleotide polymorphisms (SNPs), has revolutionized breeding programs by enabling empirical estimation of genetic relationships [6]. Genomic relationship matrices offer an alternative to traditional pedigree-based methods, improving the precision of genetic evaluations [7]. However, large-scale genotyping remains costly and complex, particularly for breeding populations over multiple generations. Hybrid approaches, such as single-step genomic BLUP (ssGBLUP), combine genomic and pedigree-based relationship matrices to enhance predictive power [8, 9]. Despite these advances, there are several methodological and computational challenges in optimizing prediction models that effectively balance genomic and pedigree information [10].

Machine learning (ML) methods have emerged as powerful alternatives to traditional linear models for genomic prediction [11, 12]. Regularized regression techniques, such as ridge regression (RR), least absolute shrinkage and selection operator (LASSO), and elastic net (EN), address the high-dimensionality of genomic data by penalizing model complexity and selecting informative markers [13–15]. These models have been extended in a range of directions [16] and have been shown to perform well on genome-wide data [17]. Random forest (RF), gradient boosting machines (GBM) and reproducing kernel Hilbert space (RKHS) methods also seem to work well in various GWP tasks, but there is no method that stands out as a clear winner in all situations [18–20].

Deep learning (DL) has gained prominence in genomic prediction due to its ability to capture complex, non-linear hidden structures in data [21, 22]. Convolutional neural networks (CNNs) are designed to extract local patterns from genomic sequences [23], while recurrent neural networks (RNNs) model sequential dependencies over chromosomes [24]. More recently, biologically interpretable neural networks have been proposed to enhance genomic prediction by incorporating structured prior knowledge. Some studies have demonstrated the potential of neural networks to predict phenotypes using multiomics data [25], while other studies emphasized the importance of efficient variable selection to identify influential genetic variants [26]. These studies highlight the importance of model interpretability and explainability, aligning with a general trend in the genomic ML and DL literature [27–29].

Recent research has investigated attention-based DL models for genomic prediction, demonstrating their ability to dynamically weight informative markers [30]. An attention mechanism is a technique that directs DL models to prioritize the most relevant parts of input data and serves as a key element of the transformer architecture [31]. Integrating attention mechanisms in genomic prediction models can improve the interpretability of features and facilitate biological insights [32]. The softmax activation function is widely recognized for its usefulness in attention mechanisms. It guarantees that the attention weights remain positive, which facilitates the model to assign significance to various input elements. The normalization constraint makes sure that the attention weights for all input elements sum to 1, rendering the weights interpretable as probabilities. In addition, the induced sparsity helps the model focus on a few key elements [33].

Multi-task learning (MTL) has been explored in genomic prediction to leverage shared information across multiple traits or environments [34]. Bayesian MTL frameworks [35], proximal MTL algorithms [36] and DL-based MTL models [37] have demonstrated satisfactory predictive performance by capturing inter-trait correlations, which is particularly valuable in breeding programs where multiple phenotypic traits are simultaneously evaluated. In DL applications, it is important to select a loss function that jointly minimizes the error over tasks without giving too much weight to certain tasks [38, 39].

In this study, we propose GRVSNN, a deep learning-based genomic prediction approach that leverages gated residual (GR) and variable selection (VS) layers within a deep learning multi-task learning framework. Our main contributions are: (1) Development of a DL-based multi-task genomic prediction model that integrates GR and VS layers, enabling adaptive feature selection and enhanced interpretability. (2) Incorporation of information from genomic markers and reduced rank principal components from pedigree-based relationship matrices into the model, allowing hybrid information for more robust predictions. (3) Evaluation of GRVSNN on real-world genomic datasets, including mouse, pig, and loblolly pine, and comparison of its performance with traditional Bayesian linear genomic prediction models (Bayesian Lasso (BLasso) [40], Bayesian ridge regression (BRR) [41], and BayesC$\pi$ [42]), as well as LassoNet [43], which recently was shown to provide a state-of-the-art approach for GWP when compared to a range of other ML and DL methods [44].

## Results

**Table 1:** Comparison of model performance on the Loblolly pine data (trait 9 and trait 10) for multi-task genomic prediction

Trait	Model	Genomic markers			Genomic markers and pedigree loadings		
		MSE (mean ± SD)	*r* (mean ± SD)	# Feature selected	MSE (mean ± SD)	*r* (mean ± SD)	# Feature selected (markers/loadings)
Trait 9	GRVSNN	**0.231 ± 0.027**	**0.683 ± 0.061**	**127**	**0.203 ± 0.033**	**0.696 ± 0.001**	**97 (55/42)**
	LassoNet	0.236 ± 0.061	0.676 ± 0.033	137	0.221 ± 0.056	0.693 ± 0.062	112 (59/53)
	BLasso	0.256 ± 0.017	0.517 ± 0.023	237	0.245 ± 0.041	0.575 ± 0.006	198 (113/85)
	BRR	0.255 ± 0.006	0.530 ± 0.055	261	0.244 ± 0.011	0.576 ± 0.007	237 (139/98)
	BayesC$\pi$	0.263 ± 0.011	0.481 ± 0.021	187	0.251 ± 0.006	0.552 ± 0.031	161 (97/64)
Trait 10	GRVSNN	**0.279 ± 0.034**	**0.656 ± 0.054**	**88**	**0.246 ± 0.051**	**0.674 ± 0.053**	**88 (43/45)**
	LassoNet	0.281 ± 0.053	0.641 ± 0.021	93	0.247 ± 0.045	0.661 ± 0.002	93 (51/42)
	BLasso	0.293 ± 0.014	0.621 ± 0.004	182	0.261 ± 0.017	0.640 ± 0.017	182 (95/87)
	BRR	0.290 ± 0.007	0.624 ± 0.031	205	0.257 ± 0.031	0.642 ± 0.004	205 (107/98)
	BayesC$\pi$	0.330 ± 0.024	0.566 ± 0.027	157	0.277 ± 0.015	0.594 ± 0.022	157 (87/70)

**Bold** indicates the best results

**Table 2:** Comparison of model performance on the mouse data for the multi-task genomic prediction

Trait	Model	Genomic markers			Genomic markers and pedigree loadings		
		MSE (mean ± SD)	*r* (mean ± SD)	# Feature selected	MSE (mean ± SD)	*r* (mean ± SD)	# Feature selected (markers/loadings)
Trait 1	GRVSNN	**0.136 ± 0.037**	**0.691 ± 0.004**	**70**	**0.131 ± 0.002**	**0.712 ± 0.034**	**64 (35/29)**
	LassoNet	0.137 ± 0.026	0.683 ± 0.007	72	0.132 ± 0.001	0.697 ± 0.021	66 (39/27)
	BLasso	0.145 ± 0.027	0.517 ± 0.033	137	0.136 ± 0.021	0.537 ± 0.005	119 (63/56)
	BRR	0.141 ± 0.005	0.525 ± 0.017	148	0.136 ± 0.017	0.547 ± 0.005	157 (87/70)
	BayesC$\pi$	0.157 ± 0.024	0.467 ± 0.023	124	0.143 ± 0.015	0.484 ± 0.007	114 (64/50)
Trait 2	GRVSNN	**0.134 ± 0.051**	**0.681 ± 0.062**	**60**	**0.133 ± 0.055**	**0.693 ± 0.005**	**60 (33/27)**
	LassoNet	0.135 ± 0.032	0.677 ± 0.032	63	0.134 ± 0.012	0.689 ± 0.037	63 (40/23)
	BLasso	0.147 ± 0.013	0.511 ± 0.026	116	0.141 ± 0.021	0.527 ± 0.017	116 (71/45)
	BRR	0.146 ± 0.015	0.517 ± 0.011	153	0.141 ± 0.022	0.533 ± 0.031	153 (85/68)
	BayesC$\pi$	0.151 ± 0.028	0.455 ± 0.018	105	0.146 ± 0.031	0.471 ± 0.015	105 (63/42)

**Bold** indicates the best results

**Table 3:** Comparison of model performance on the pig data for the multi-task genomic prediction

Trait	Model	Genomic markers			Genomic markers and pedigree loadings		
		MSE (mean ± SD)	*r* (mean ± SD)	# Feature selected	MSE (mean ± SD)	*r* (mean ± SD)	# Feature selected (markers/loadings)
Trait 1	GRVSNN	**0.145 ± 0.003**	**0.653 ± 0.005**	**187**	**0.131 ± 0.004**	**0.711 ±0.033**	**147 (72/75)**
	LassoNet	0.147 ± 0.005	0.641 ± 0.016	195	**0.131 ± 0.031**	0.702 ± 0.002	155 (83/72)
	BLasso	0.154 ± 0.024	0.443 ± 0.07	273	0.137 ± 0.041	0.515 ± 0.005	177 (91/86)
	BRR	0.152 ± 0.011	0.442 ± 0.021	297	0.136 ± 0.005	0.533 ± 0.006	207 (119/88)
	BayesC$\pi$	0.157 ± 0.051	0.417 ± 0.004	205	0.147 ± 0.017	0.463 ± 0.012	162 (93/69)
Trait 2	GRVSNN	**0.147 ± 0.047**	**0.652 ± 0.005**	**179**	**0.133 ± 0.017**	**0.704 ± 0.005**	**132 (67/55)**
	LassoNet	0.151 ± 0.023	0.649 ± 0.007	181	0.134 ± 0.039	0.685 ± 0.008	144 (84/60)
	BLasso	0.158 ± 0.023	0.427 ± 0.007	267	0.137 ± 0.036	0.465 ± 0.005	176 (92/84)
	BRR	0.155 ± 0.007	0.433 ± 0.006	285	0.135 ± 0.002	0.480 ± 0.013	203 (107/96)
	BayesC$\pi$	0.165 ± 0.007	0.424 ± 0.005	197	0.144 ± 0.037	0.437 ± 0.005	151 (82/79)
Trait 3	GRVSNN	**0.143 ± 0.006**	**0.701 ± 0.006**	**189**	**0.132 ± 0.068**	**0.725 ± 0.006**	**135 (71/64)**
	LassoNet	0.151 ± 0.002	0.680 ± 0.011	191	0.133 ± 0.049	0.703 ± 0.007	141 (81/60)
	BLasso	0.164 ± 0.023	0.564 ± 0.007	277	0.143 ± 0.004	0.627 ± 0.005	170 (87/83)
	BRR	0.161 ± 0.007	0.565 ± 0.004	296	0.142 ± 0.006	0.628 ± 0.007	195 (97/88)
	BayesC$\pi$	0.175 ± 0.022	0.495 ± 0.004	203	0.154 ± 0.037	0.531 ± 0.002	154 (81/73)
Trait 4	GRVSNN	**0.136 ± 0.005**	**0.611 ± 0.006**	**187**	**0.131 ± 0.011**	**0.632 ± 0.005**	**140 (73/67)**
	LassoNet	0.138 ± 0.003	0.602 ± 0.007	195	0.132 ± 0.022	0.631 ± 0.003	149 (79/70)
	BLasso	0.145 ± 0.023	0.457 ± 0.005	205	0.136 ± 0.004	0.529 ±0.005	177 (91/86)
	BRR	0.143 ± 0.007	0.477 ± 0.007	249	0.136 ± 0.002	0.536 ± 0.008	205 (110/95)
	BayesC$\pi$	0.155 ± 0.037	0.410 ± 0.005	193	0.143 ± 0.032	0.511 ± 0.006	151 (80/71)
Trait 5	GRVSNN	**0.141 ± 0.007**	**0.607 ± 0.005**	**191**	**0.135 ± 0.013**	**0.639 ± 0.004**	**153 (79/74)**
	LassoNet	0.142 ± 0.001	0.603 ± 0.007	203	0.136 ± 0.003	0.621 ± 0.019	171 (89/82)
	BLasso	0.158 ± 0.033	0.467 ± 0.001	237	0.141 ± 0.021	0.511 ± 0.017	203 (107/96)
	BRR	0.157 ± 0.003	0.501 ± 0.005	252	0.140 ± 0.033	0.534 ± 0.005	209 (116/93)
	BayesC$\pi$	0.161 ± 0.016	0.427 ± 0.024	217	0.151 ± 0.013	0.437 ± 0.023	177 (91/86)

**Bold** indicates the best results for each trait

**Table 4:** Comparison of distance correlation (dCor) across models and datasets for multi-task genomic prediction

Dataset	Data	Trait	GRVSNN	LassoNet	BLasso	BRR	BayesC$\pi$
Loblolly pine data	Genomic markers	Trait 9	**0**.**833**	0.801	0.800	0.770	0.783
		Trait 10	**0**.**490**	0.445	0.411	0.462	0.432
	Genomic markers and pedigree loadings	Trait 9	**0**.**874**	0.840	0.839	0.830	0.803
		Trait 10	**0**.**498**	0.465	0.421	0.473	0.442
Mouse data	Genomic markers	Trait 1	**0**.**730**	0.708	0.654	0.645	0.611
		Trait 2	**0**.**251**	0.215	0.154	0.129	0.129
	Genomic markers and pedigree loadings	Trait 1	**0**.**732**	0.710	0.672	0.660	0.624
		Trait 2	**0**.**310**	0.235	0.179	0.175	0.158
Pig data	Genomic markers	Trait 1	**0**.**891**	0.890	0.862	0.853	0.810
		Trait 2	**0**.**912**	0.910	0.882	0.888	0.812
		Trait 3	**0**.**901**	0.893	0.886	0.895	0.877
		Trait 4	**0**.**971**	0.964	0.886	0.895	0.810
		Trait 5	**0**.**923**	0.910	0.902	0.915	0.865
	Genomic markers and pedigree loadings	Trait 1	**0**.**903**	0.900	0.881	0.882	0.835
		Trait 2	**0**.**923**	0.917	0.892	0.904	0.832
		Trait 3	**0**.**909**	0.899	0.890	0.898	0.880
		Trait 4	**0**.**983**	0.981	0.961	0.962	0.928
		Trait 5	**0**.**943**	0.930	0.913	0.924	0.876

**Bold** indicates the best result for each trait

**Fig. 1:**
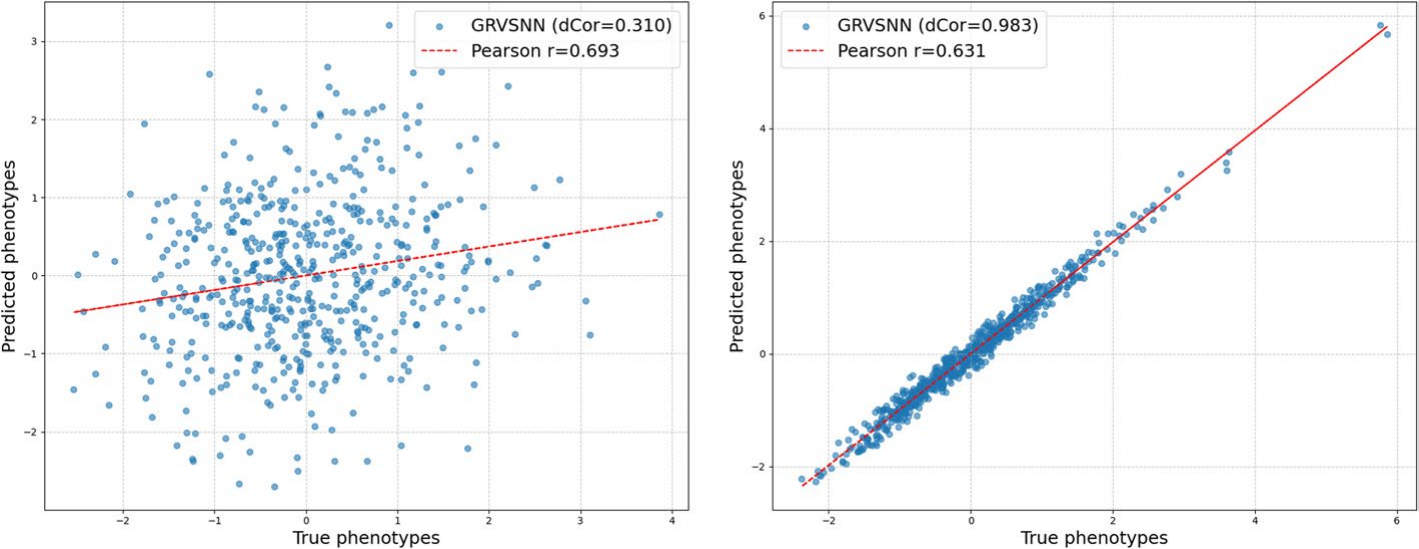
True phenotypes vs. predicted phenotypes from GRVSNN analyses of data with genomic markers and pedigree loadings. Pearson (*r*) and distance (dCor) correlations are reported. Left: Trait 2 from the mouse data, right: Trait 4 from the pig data

### Model comparison

#### Loblolly pine data

Tabel 1 presents the experimental results obtained from the loblolly pine data across various models. We can clearly see that the proposed GRVSNN model consistently outperforms the three Bayesian approaches as well as LassoNet. When utilizing genomic data alone, GRVSNN achieves mean test MSEs of 0.231 for Trait 9 and 0.279 for Trait 10. In contrast, the Bayesian methods yield higher error rates: BayesC$\pi$ results in MSEs of 0.263 and 0.330; BRR yields 0.255 and 0.290; and BLasso yields 0.256 and 0.293 for Traits 9 and 10, respectively. LassoNet produces improved performance over the Bayesian baselines with MSEs of 0.236 (Trait 9) and 0.281 (Trait 10), yet it still underperforms relative to GRVSNN. Notice that the optimal hyperparameters for LassoNet in these experiments were a feature budget $M=26.7$ and a regularization strength factor of $\lambda =15$. Incorporating pedigree-based loading further enhances the performance of GRVSNN, reducing the test MSE to 0.203 for Trait 9 and 0.246 for Trait 10. These results correspond to relative improvements of approximately 12.1% for Trait 9 and 11.8% for Trait 10, respectively, demonstrating the effectiveness of integrating supportive pedigree information into GWP.

We can also note in Table 1 that the Pearson correlation coefficient (*r*) supports the superior predictive performance of the suggested GRVSNN model. With only genomic data, *r* reaches 0.683 for Trait 9 and 0.656 for Trait 10, exceeding the performance of other models such as BLasso ($r = 0.517$ for Trait 9 and $r = 0.621$ for Trait 10), BRR ($r = 0.530$ for Trait 9 and $r = 0.624$ for Trait 10) and BayesC$\pi$ ($r = 0.481$ for Trait 9 and $r = 0.566$ for Trait 10). The inclusion of pedigree loadings improves the *r*-values for GRVSNN to 0.696 for Trait 9 and to 0.674 for Trait 10.

In Table 4, we can see that GRVSNN consistently achieves the highest dCor estimates across both traits and experimental settings. Using only genomic markers, GRVSNN attains dCor values of 0.833 (Trait 9) and 0.490 (Trait 10), outperforming LassoNet and all Bayesian baseline methods. Incorporating pedigree loadings further improves GRVSNN’s dCor to 0.874 and 0.498, respectively. These improvements reflect GRVSNN’s enhanced ability to capture non-linear and multi-trait dependencies.

#### Mouse data

Table 2 reports the experimental results for different models applied over the mouse data. We can observe that the GRVSNN method consistently outperforms all other methods in both predictive accuracy and feature selection efficiency. For Trait 1, GRVSNN achieves the lowest MSE (0.136) when using only genomic markers, surpassing LassoNet (MSE = 0.137), BLasso (MSE = 0.145), BRR (MSE = 0.141) and BayesC$\pi$ (MSE = 0.157). When incorporating pedigree loadings, GRVSNN further improves its prediction MSE to 0.131, corresponding to a relative improvement of 3.7%, while maintaining the smallest number of features selected (64 selected features).

The trend is similar for Trait 2, where GRVSNN achieves the best performance, with MSE of 0.134 in the genomic-only setting and 0.133 when including pedigree loadings. LassoNet ($M=20.5$ and $\lambda =35$) produces similar MSE values (0.135 and 0.134, respectively), but the Bayesian models clearly show higher MSE values. The results for the Pearson correlation coefficient (*r*) further confirm these patterns. For Trait 1, GRVSNN achieves an *r* of 0.691 with genomic data alone, improving to 0.712 with genomic loadings. Similarly, for Trait 2, GRVSNN obtains an *r* of 0.681 and 0.693, respectively, outperforming all other models.

Regarding the dCor comparison, we can note for Trait 1 that GRVSNN achieves 0.730 with genomic markers which improves slightly to 0.732 when adding pedigree loadings. For Trait 2, GRVSNN delivers dCor estimates of 0.251 without loadings and 0.310 with loadings which is considerably higher than the Bayesian models that show considerably lower dCor values. This indicates that GRVSNN is better at uncovering complex dependency patterns also of the mice traits than other methods.

#### Pig data

Table 3 details the predictive performance of different models in the pig data across five traits. The results demonstrate that the proposed GRVSNN model maintains its superior predictive performance across all five traits on the pig data in terms of both test MSE and Pearson correlation coefficient (*r*). For Trait 1, GRVSNN achieves the lowest MSE of 0.145 using genomic markers alone, further improving to 0.131 with the inclusion of pedigree loadings. This represents an significant improvement of approximately 9.6%, highlighting the advantage of incorporating additional latent structures. For the Trait 2, GRVSNN reduces MSE from 0.147 to 0.133 when the pedigrees information is included. The Pearson correlation coefficient (*r*) further supports the superior predictive capability of GRVSNN with a clear improvement from 0.652 to 0.704. In terms of trait 3, it achieves an *r* of 0.701 with genomic markers alone, improving to 0.725 with pedigree loadings. For Trait 4, GRVSNN continues to outperform its competitors, reducing MSE to 0.136 and achieving an *r* of 0.611 with genomic markers alone, which improves to $r=0.632$ with pedigree information. LassoNet follows GRVSNN closely, but with a higher test MSE of 0.151 and 0.134, and with lower correlation coefficients. At the same time, we see that LassoNet ($M=18.3$ and $\lambda =47$) selects a slightly higher number of features. The Bayesian models produce considerably lower correlation values and higher MSE, reinforcing their weaker predictive strength. Trait 5 further demonstrates GRVSNN’ efficiency, as it attains the best performance with an MSE of 0.141 and an *r* of 0.607, further improving to an MSE of 0.135 and an *r* of 0.639 when pedigree loadings are included. In comparison, LassoNet, the second-best performer, achieves *r* of 0.680 and 0.703 in the respective settings. The Bayesian methods exhibit significantly lower correlation values and higher test MSE also on this trait.

For the pig data, GRVSNN achieves strikingly high dCor values across all five traits (Table 4). Using only genomic markers, it reaches 0.971 for Trait 4, with the next-best model, LassoNet, slightly behind at 0.964. With the addition of pedigree loadings, GRVSNN further improves dCor to 0.983 on Trait 4, delivering consistent top performance across all traits and demonstrating its superior capability in capturing the strength of association. Figure 1 provides plots of the observations *y* and predicted values ${\hat{y}}$ for GRVSNN of trait 2 from the mice data (left) and trait 4 from the pig data (right), showing that dCor provides a better tool than *r* to investigate the strengths of associations.

### Interpretability analysis

Interpretability has become an important factor in genomic prediction models. The proposed GRVSNN incorporates variable selection into the network architecture through the sparsity mechanisms imposed in both the input and latent feature layers. The important mechanism enabling feature selection is the local reweighted feature importance learned via the sigmoid gating procedure and the global softmax operation. The network effectively sets the weights of less informative variables to zero or to small values close to zero, ensuring that only the most relevant features are retained. By comparison, LassoNet performs variable selection using a hierarchical sparsity constraint enforced by a residual connection between the input and hidden layers. It applies a path-regularized Lasso penalty, which ensures that only the most predictive features remain active while less important ones are removed as their weights shrink to zero. In contrast, variable selection in the Bayesian models we have used is obtained via MCMC sampling techniques by calculating $95\%$ credible intervals (CIs) of the regression parameter estimates and checking if the CIs overlap zero.

The differences in interpretability across models are shown in Tables 1,  2, and  3. In the loblolly pine dataset, GRVSNN selects the smallest subset of features, identifying 127 markers for Trait 9 and 88 for Trait 10 in the genomic-only setting. When incorporating pedigree loadings, the feature set is further refined to 97 (55 markers and 42 loadings) for Trait 9 and to 88 (43 markers and 45 loadings) for Trait 10. Although LassoNet is competitive, it selects slightly more features (e.g., 137 markers for Trait 9 and 93 for Trait 10), whereas the Bayesian models retain more than 200 features. For the mouse data, GRVSNN maintains a better interpretability advantage by identifying only 70 features in the genomic-only setting, reducing to 64 when incorporating pedigree loadings (35 markers and 29 loadings) for Trait 1. Similarly, for Trait 2, it selects 60 features (33 markers and 27 loadings). The Bayesian methods, on the contrary, select over 100 features, leading to reduced model interpretability. Finally, in the pig data, GRVSNN selects the smallest number of markers for all traits in both the genomic-only and combined genomic plus loadings settings. LassoNet selects slightly more features, while the Bayesian models selects more than 250 features. These findings highlight that GRVSNN effectively balance good predictive performance with sparsity, making it a robust choice for genomic prediction tasks where both accuracy and feature selection are essential.

### Computing time comparison

**Table 5:** Time report in minutes (m) (mean ± SD) across datasets and models for different models (fivefold cross-validation)

Model	Loblolly pine data		Mouse data		Pig data	
	Genomic markers	Genomic markers and pedigree loadings	Genomic markers	Genomic markers and pedigree loadings	Genomic markers	Genomic markers and pedigree loadings
GRVSNN	**6.1 ± 0.012**	**3.4 ± 0.015**	**8.4 ± 0.003**	**5.1 ± 0.006**	**11.3 ± 0.004**	**5.7 ± 0.015**
LassoNet	10.3 ± 0.004	6.4 ± 0.007	15.4 ± 0.003	10.2 ± 0.004	20.5 ± 0.002	11.3 ± 0.003
BLasso	13.5 ± 0.004	8.5 ± 0.003	14.2 ± 0.004	7.6 ± 0.007	22.1 ± 0.006	13.7 ± 0.005
BRR	13.1 ± 0.005	8.7 ± 0.006	16.3 ± 0.003	8.2 ± 0.005	19.5 ± 0.003	9.8 ± 0.002
BayesC$\pi$	13.5 ± 0.004	7.8 ± 0.007	15.1 ± 0.004	8.1 ± 0.006	20.3 ± 0.006	11.4 ± 0.006

To evaluate the computational efficiency of different methods, we compare the training times across our three datasets in two settings: using only genomic markers and jointly incorporating genomic markers with pedigree loadings. Table 5 presents the average runtime (in minutes) for GRVSNN (with a hard-sigmoid function), LassoNet, BLasso, BRR, and BayesC$\pi$ across all datasets using five-fold cross-validation.

The results in Table 5 indicate that the pig dataset exhibits the longest runtime across all models, due to its larger sample size and higher feature dimensionality. In contrast, the loblolly pine dataset has the lowest runtime, reflecting its smaller number of individuals and features. Across all methods, incorporating genomic markers with pedigree loadings significantly reduces runtime compared to using only genomic markers. Among all models, GRVSNN achieve the lowest computational time across datasets, particularly when integrating genomic markers with pedigree loadings. For instance, in the loblolly pine dataset, the runtime decreases from 6.1 to 3.4 min. A similar trend is observed for the mice and pig datasets, where GRVSNN consistently outperform other methods in runtime efficiency. LassoNet, which employs two hidden layers in its feedforward network, also benefits from dimensionality reduction via pedigree loadings, but remains slower than GRVSNN. For instance, on the pig dataset, its runtime decreases from 20.5 to 11.3 min when using genomic markers with pedigree loadings, yet it remains considerably higher than GRVSNN’ 5.7 min. The Bayesian methods (BLasso, BRR, and BayesC$\pi$) generally exhibit longer runtimes across all datasets due to their reliance on Markov Chain Monte Carlo (MCMC) sampling. Also with pedigree loadings, these methods remain significantly slower than GRVSNN and LassoNet. We can notice that the timing varies slightly between the Bayesian methods, but there is no clear best method. Finally, we need to mention that the number of MCMC iterations of course influences the timings of the Bayesian methods. We have chosen 6000 MCMC iterations which definitely can be seen as a minimum that probably should be increased at least 5-fold to obtain better mixing and effective sample sizes.

Furthermore, GRVSNN exhibit improved convergence speed due to the use of the hard-sigmoid activation function in the gated residual block. Specifically, convergence occurs at 71 iterations for loblolly pine data, 97 iterations for mouse data, and 137 iterations for pig data with the hard-sigmoid function, compared to 85, 117, and 163 iterations, respectively, with the soft-sigmoid function. We also evaluated timings of switching the softmax activation function to the sparsemax activation function, but no improvements were obtained in that case.

## Discussion

In this study, we have proposed a multi-task deep learning approach to genomic prediction, referred to as the gated residual variable selection neural networks (GRVSNN). Unlike most other DL models, our method effectively performs feature selection on input data, enhancing both interpretability and predictive accuracy. GRVSNN is a tabular method and we illustrate the ease of data integration by incorporation of features from a reduced-rank transformation of polygenic pedigrees. By using a gated residual block followed by a variable selection block, the model can learn task-specific non-linear transformations and feature importance. Hence, the proposed GRVSNN enable both local feature-specific and global variable selection. The results from three datasets demonstrate the robustness and computational efficiency of the GRVSNN framework.

A crucial aspect of genomic prediction is the reliable assessment of model performance. While traditional metrics like Mean Squared Error (MSE) and Pearson correlation coefficient (*r*) are widely used, they may not fully capture the complex, often non-linear relationships inherent in genomic data. To provide a more nuanced statistical assessment, we chose to complement the Pearson correlation coefficient with the distance correlation coefficient (dCor) [45]. Unlike Pearson’s *r*, which exclusively measures linear associations, dCor is a model-free, nonparametric measure capable of detecting both linear and non-linear dependencies between two random vectors. This makes dCor particularly suitable for genomic prediction tasks where intricate and non-linear genetic architectures are expected. Our analyses, summarized in Table 4, consistently show that GRVSNN achieves the highest dCor values across all evaluated datasets and traits. For instance, in the pig data, GRVSNN reaches a dCor of 0.9827 for Trait 4 with markers and pedigree loadings. On the other hand, the dCor value of trait 2 from the mice data is 0.310 which is considerably lower. Figure 1 visually illustrates this difference between the true and predicted phenotypes, demonstrating the ability of dCor to better capture different association strengths. Another important point is that the inherent bias-variance trade-off in predictive modeling and the non-i.i.d. nature of predicted values, make standard statistical tests (e.g., t-tests on MSE or correlation coefficients) inappropriate for evaluating these machine learning outcomes. Thus, reporting dCor estimates provides a more reliable and statistically justified measure of overall association without violating basic statistical assumptions.

Bayesian methods have been widely used for genomic prediction due to their ability to perform adaptive regularization. These methods, for example BLasso, BRR, and BayesC$\pi$, perform regularization via the prior distributions by shrinking irrelevant coefficients while allowing large effects for informative markers. One should also keep in mind that the these Bayesian methods need some form of post-hoc analysis to evaluate sparsity. We have checked if the $95\%$ CIs of the regression coefficients overlap zero for all input features because this is the most straightforward approach using the BGLR package. More advanced and efficient Bayesian regularization and variable selection approaches include those based on global–local shrinkage priors [46]. However, these models often struggle with computational efficiency in high-dimensional settings [47], making them impractical for large genomic datasets. Our empirical results on the different datasets confirm this limitation, as the Bayesian models consistently demonstrate longer computation times and lower predictive performance. In contrast, LassoNet, which incorporates structured sparsity through a hierarchical grouping mechanism, has shown competitive predictive accuracy while maintaining computational efficiency compared to other tabular DL methods and gradient boosting machines [44]. Compared to the Bayesian approaches that we evaluated in our study, LassoNet achieves better prediction performance at the same time as it selects a smaller subset of features. However, the GRVSNN framework surpasses LassoNet in terms of predictive performance, sparsity, and lower computational demand, particularly when incorporating pedigree information.

Recent advances in transformer-based models and genomic language models (gLMs) have demonstrated impressive performance in capturing long-range dependencies in genomic data [30]. However, their extreme computational demands remain a significant barrier to widespread adoption. Although transformers have achieved state-of-the-art results in various genomic tasks, training and deploying these models require substantial hardware resources, limiting their feasibility in many practical genomic prediction applications. This computational bottleneck highlights the trade-off between model complexity and scalability, positioning GRVSNN as a more balanced approach that effectively integrates genomic and pedigree data while maintaining computational feasibility. Some efforts have explored alternative neural network architectures to circumvent the excessive parameterization and computational burden of transformer-based gLMs. For example, multi-layer perceptron (MLP)-based architectures as MLP-Mixer and related variations have demonstrated promising results in tabular and genomic prediction tasks [48, 49]. These models leverage structured sparsity and channel-mixing operations to improve predictive power. The advancements in these MLP-based models suggest that it is possible to enhance genomic prediction models without resorting to computationally expensive transformer-based solutions. GRVSNN align with this trend, as it shares the goal of balancing expressivity and efficiency by incorporating specialized feature selection and residual learning mechanisms.

Despite its superior performance, GRVSNN has some limitations: (1) Although it outperforms LassoNet and the Bayesian methods in training efficiency, it still demands considerable computational GPU resources for tuning of parameters, for example optimization of learning rate, dropout rate, and network depth to achieve optimal results. (2) GRVSNN can only be trained on specific datasets, limiting the generalization to new genetic backgrounds. Unlike gLMs [50], which uses large-scale pretraining on genomic sequences, GRVSNN lack such inductive transferability, potentially restricting their applicability across species. Future work could explore hybrid models that incorporate both GRVSNN and efficient MLP-based designs to further optimize genomic prediction models.

## Conclusion

The gated residual variable selection neural networks (GRVSNN) introduces a novel multi-task genomic prediction framework which integrates pedigree-based low-rank information with genomic markers to improve predictive accuracy and interpretability. By combining gated residual blocks and variable selection blocks, GRVSNN effectively selects a small number of informative genetic markers, improving biological interpretability while maintaining computational efficiency. The experimental results from evaluation on mouse, pig, and loblolly pine datasets demonstrate that GRVSNN consistently outperforms traditional Bayesian regression models (BLasso, BRR, and BayesC$\pi$) and LassoNet by achieving lower test prediction error and higher Pearson correlation (*r*) and distance correlation (dCor) coefficients. In addition, incorporation of pedigree loadings significantly improves prediction accuracy and reduces computational cost, further enhancing the suitability of GRVSNN for large-scale genomic studies.

## Methods

This section describes the methodology for genomic prediction and variable selection using the deep learning-based GRVSNN framework. Our approach is designed to efficiently handle high-dimensional genomic data by improving feature selection and pedigree transformation, leveraging multi-task learning for enhanced predictive performance and interpretability across multiple traits.

### Reduced-rank transformation of the polygenic pedigree

Statistical modeling with genetic covariance matrices is essential to understand the genetic architecture of complex traits [51]. Traditional estimation methods often fail when the number of traits exceeds the number of individuals, leading to unstable estimates, noise, and spurious correlations that obscure true genetic relationships [52]. Reduced-rank estimation provides a robust alternative by stabilizing estimates, reducing noise, and preserving key genetic components while maintaining a parsimonious structure [53]. This reduced-rank approach is particularly useful in applications such as animal and plant breeding, where optimizing multiple traits simultaneously requires reliable genetic correlation estimates [7].

The pedigree matrix *A* is first decomposed using a traditional eigendecomposition, $A=Q\Lambda Q^{-1}$ where *Q* denotes the eigenvectors and $\Lambda$ contains the eigenvalues. The loadings *L* are then calculated as:

1 $$\begin{aligned} L = Q \times \sqrt{\Lambda }. \end{aligned}$$

After that, the eigenvalues are sorted in descending order and used in a scree plot to determine where the eigenvalues are no longer decreasing [54]. This rank value is the reduced rank *m* that is used to obtain the reduced rank loadings matrix $L_r$. We integrate these reduced-rank loadings into the genomic prediction framework by combining them with the genomic markers *M* into a joint tabular input matrix $X=[L_r,M]$.

### Gated residual block

The GR block consists of multiple layers designed to facilitate feature transformation while it effectively controls information flow. The input features, $X \in {\mathbb {R}}^{P}$, where *P* is the total number of features, are one by one first submitted to a non-linear layer:

2 $$\begin{aligned} f_1 = \sigma _{f}(W_{1} x + b_{1}), \end{aligned}$$

where $W_{1}$ is a learnable weight matrix, $b_{1}$ is the bias term and $\sigma _{f}$ is the exponential linear unit (ELU) activation function. The ELU function with $\gamma > 0$ is defined as:

3 $$\begin{aligned} \text{ELU}(x) = {\left\{ \begin{array}{ll} x, & \text{if } x>0 \\ \gamma (e^{x} - 1), & \text{if } x \le 0 \end{array}\right. }, \end{aligned}$$

where $\gamma$ is a positive constant that controls the saturation point for negative values. We use the default value $\gamma =1$. Unlike the ReLU active function, which set all negative inputs to zero, ELU allows small negative outputs, which is helpful to push the mean activations closer to zero, improving convergence speed and reducing bias shift [55]. Following the non-linear transformation, a linear layer with dropout is applied to prevent overfitting that yields an output $f_2$, which is connected to a gated linear unit:

4 $$\begin{aligned} h = \sigma _{h}(W_{2} f_2 + b_{2}), \end{aligned}$$

where $W_{2}$ is another learnable weight matrix, $b_{2}$ is the bias term and $\sigma _{h}$ is the linear activation function. To regulate information flow, a gating mechanism is then introduced as:

5 $$\begin{aligned} g = \sigma _{g}(W_{3} f_2 + b_{3}), \end{aligned}$$

where $W_{3}$ is another learnable weight matrix, $b_{3}$ is the bias term, and $\sigma _{g}$ is the sigmoid (or hard sigmoid) activation function. The gating vector $g \in (0, 1)^{n_{h}}$ determines how much of the transformed features contribute to the final output. A residual connection is then incorporated to stabilize training and retain original input information as:

6 $$\begin{aligned} z = g \odot h + (1 - g) \odot x, \end{aligned}$$

where $\odot$ represents element-wise multiplication. This operation allows the model to learn which input features should be set to zero and therefore be canceled out. The final output is normalized using layer normalization as:

7 $$\begin{aligned} {\hat{z}} = \text{LayerNorm}(z). \end{aligned}$$

Note that layer normalization ensures stable training by normalizing activations across feature dimensions, mitigating internal covariate shift. The output ${\hat{z}}$ is then passed to subsequent shared layers for further processing. The GR blocks are employed locally on each input but share information over inputs and tasks via the subsequent VS block and multitask loss function.

### Variable selection block

The VS block is used to identify the most important features globally across all inputs. This addresses the challenge of selecting a subset of informative genomic and pedigree loading inputs that contribute to the phenotypes. The importance of each transformed feature ${\hat{z}}_{i}$ is determined via a softmax function:

8 $$\begin{aligned} w_i = \frac{e^{{\hat{z}}_{i}}}{\sum _j e^{{\hat{z}}_{j}}}, \end{aligned}$$

where $w_{i}$ represents the relative importance of feature ${\hat{z}}_{i}$. The softmax function ensures that the weights are non-negative and sum to one, allowing for a probabilistic interpretation. The selected feature subset is then obtained by re-weighting the input features as:

9 $$\begin{aligned} z^{'}_{i} = w_{i} \cdot {\hat{z}}_{i}. \end{aligned}$$

This operation can adaptively scale the features, enhancing the influence of more relevant ones while suppressing others. Features with higher weights are retained with greater influence, while those with lower weights are down-weighted. The transformed features $z^{'}_{i}$ are then used to obtain a multi-task prediction $\hat{{\textbf{Y}}}$ for *N* individuals and and *T* traits. The model is trained by minimizing the joint multivariate MSE loss function over all traits:

10 $$\begin{aligned} {\mathcal {L}}_{\text{MSE}} = \left\| {\textbf{Y}} - \hat{{\textbf{Y}}} \right\| _{2}^{2}. \end{aligned}$$

By combining the GR and VS blocks, the proposed framework performs both local feature-specific and global variable selection. This approach parallels the functionality of global–local shrinkage priors commonly used in linear Bayesian variable selection methods [46]. The gated residual (GR) block combines residual connections, which preserve linear additive genetic effects, with nonlinear transformation layers that enable the model to capture non-additive genetic effects. Hence, the GR block extends a multi-layer perceptron (MLP), where activation functions handle non-linear dominance effects and hidden layer nodes model epistatic interactions between genomic markers. The variable selection (VS) block operates globally, reweighting features across the input space to prioritize the most informative additive and non-additive contributions. Moreover, the residual connections and gating mechanisms enable the model to focus on the important inputs, avoiding the huge number of variables typically encountered when modeling epistatic effects with linear models. Finally, the output layer integrates the selected features to produce multi-trait predictions. By distributing additive and non-additive modeling responsibilities across these distinct components, the GRVSNN framework effectively balances interpretability with predictive flexibility.

### Training procedure

**Fig. 2:**
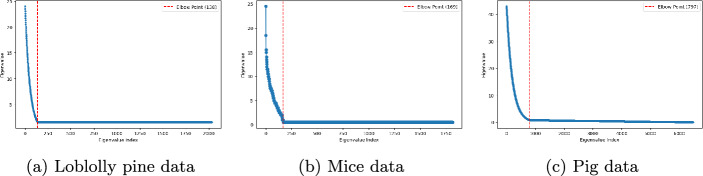
Sorted eigenvalues from the pedigree decompositions for the loblolly pine, mice and pig data. The dotted red lines indicate where the reduced rank selection were performed

To effectively train the GRVSNN model across different datasets, we begin by preprocessing the pedigree matrix *A* using dimensionality reduction. This is performed by calculation of loadings, where eigenvalues are computed, sorted, and truncated at the elbow point to retain the most informative components. Specifically, we extracted 169 components for the mice data, 797 for the pig data, and 138 for the loblolly pine data (see Fig. 2).

Then the GRVSNN model is trained using an optimization strategy that balances predictive accuracy and computational efficiency. Several hyperparameters are fine-tuned using Bayesian optimization (BO) [56], including batch size (64–512), learning rate (0.00001–0.001), and dropout rate (0.1–0.5). To prevent overfitting, early stopping is applied, terminating training if validation performance does not improve for 25 consecutive epochs. Additionally, efficiency of the BO is enhanced by dynamically monitoring the test loss: if the test loss remains stable for 30 consecutive iterations, the BO is stopped to avoid unnecessary computations while ensuring convergence. To improve regularization and sequential dependencies, we integrate the GR block with $L_2$ normalization, applied to ELU and sigmoid activations. We also evaluate the hard-sigmoid function to accelerate convergence and enhance sparsity. The hard-sigmoid function is defined as $\text{max}(0,\text{min}(1, 0.2\alpha _{i} + 0.5))$, which can ensure that values are smoothly mapped between 0 and 1 while maintaining computational efficiency and preventing vanishing gradients [57]. We also explore the sparsemax activation function as an alternative to softmax for feature selection due to its ability to enforce harder sparsity. However, empirical results showed no significant improvement over softmax in our multi-task genomic prediction tasks. Thus, we opted to retain softmax for its stable performance and easy interpretation. The model is optimized using the Adam optimizer with weight decay for additional regularization. Training is performed using 5-fold cross-validation to ensure robust performance across datasets. Specifically, the dataset is randomly partitioned into five equal folds with each fold serving once as test set and the remaining four as training set. To obtain reliable performance estimates, we repeat the 5-fold cross-validation procedure ten times using different random seeds, resulting in a total of 50 cross-validation runs per method. The final performance metrics are reported as the mean ± standard deviation across all repetitions, providing an estimate of the variability due to random partitioning. Random splits are generated using stratified sampling to preserve the distribution of trait values across folds. These combined strategies enable effective feature selection, improved generalization, and enhanced prediction accuracy in high-dimensional data. For feature selection, a threshold of 0.05 is applied to the feature importance scores $z^{'}$ in equation (9), with the number of selected features determined by counting all features whose importance values meet or exceed this predefined threshold.

### Other settings

LassoNet is implemented with two hidden layers, and its key hyperparameters - hierarchy coefficient *M* (range: 10–50) and regularization strength $\lambda$ (range: 0.0001–100) - are tuned using Bayesian Optimization (BO) over a maximum of 200 iterations. Additional parameters, such as batch size and number of nodes per layer, are also optimized via BO. A 5-fold cross-validation approach is applied to select the best parameters based on the validation MSE. The BO tuning process applies early stopping which means that it continues until a predefined stopping criterion ($1e^{-5}$) is reached. To further enhance parameters optimization, we leverage the tree-structured parzen estimator (TPE), which ensures diverse candidate suggestions across iterations while incorporating new recommendations from BO [56].

In addition to LassoNet, we also compare with three widely used traditional Bayesian regression methods: Bayesian Lasso (BLasso) [40], Bayesian ridge regression (BRR) [41], and BayesC$\pi$ [42]. BLasso assumes that marker effects follow a double-exponential (Laplace) prior, which induces a strong shrinkage toward zero for small effects that encourages sparsity. BRR assumes Gaussian priors on marker effects, leading to homogeneous shrinkage across all markers without variable selection. BayesC$\pi$ performs variable selection using a spike-and-slab mixture prior where a proportion $\pi$ of marker effects is assumed to be exactly zero while the remaining effects $(1 -\pi )$ follow a Gaussian distribution. These different priors result in different shrinkage and selection behaviors, which we benchmark against the GRVSNN framework. For these Bayesian models, test predictions are obtained via Gibbs sampling with 6000 iterations, discarding the first 2000 iterations as burn-in. Variable selection for the Bayesian methods is performed by checking if the $95\%$ credible intervals overlap zero or not. Implementation is carried out using the BGLR package in R using default parameters [58]. To ensure numerical stability and efficient convergence, all response and explanatory features are centered to zero mean and variance one before optimization, eliminating the need for an intercept term. For performance assessment, we compute the average test MSE, Pearson correlation coefficient (*r*) and distance correlation coefficient (dCor) [45] for all traits of each dataset.

## Materials

### Loblolly pine data

The loblolly pine dataset originates from 32 parents trees representing a wide range of accessions from the Atlantic coastal plain, Florida, and the lower Gulf of the United States. The parents were crossed in a circular mating design with additional off-diagonal crosses, resulting in 70 full-sib families with an average of 13.5 individuals per family [59, 60]. It was originally composed of 951 individuals from 61 families that were genotyped using an Illumina Infinium assay [61]. A subset of 4,853 SNPs (encoded as 0, 1, 2) were polymorphic and used in our study. We chose Trait 9 (rootnum) and Trait 10 (rootnumbin) for prediction because they had less than 5% missing data. After data cleaning, we retained 926 individuals for analysis. The loblolly pine pedigree structure is represented as the additive relationship matrix (*A*) [1] and the data contains a larger number of individuals (2034) in the pedigree than the number of individuals (926) with genomic markers. To retain the influence of the full pedigree matrix, we first performed eigendecomposition and then selected the eigenvectors and eigenvalues corresponding to the individuals with genomic markers. This transformation preserves both directionality (eigenvectors) and variance weighting (eigenvalues), ensuring that only the most informative components contribute to the model. The elbow point analysis of the sorted eigenvalues determined that 138 eigenvectors should be retained for this dataset (Fig. 1a). To evaluate the effect of incorporation of reduced-rank transformations, we construct two datasets: only genomic markers and combined genomic markers and pedigree loadings.

### Mouse data

The mouse dataset used in this study is sourced from the BGLR package in R [58] and originally obtained from the Wellcome Trust (http://gscan.well.ox.ac.uk). It has been widely used in several whole-genome regression studies [62, 63]. The dataset contains 10,346 single nucleotide polymorphisms (SNPs) coded as 0, 1, or 2, as well as phenotypic and pedigree information from 1,814 mice. For this study, we focus on two continuous traits: body length (BL) (Trait 1) and body mass index (BMI) (Trait 2) as response variables. Since the number of individuals in the pedigree matrix matches that in the genomic marker data, we applied eigendecomposition to *A*, extracting eigenvalues and eigenvectors to calculate pedigree loadings. The elbow point of the sorted eigenvalues was used to determine the optimal number of retained components, resulting in 169 selected eigenvectors for downstream analysis (Fig. 1b).

### Pig data

The pig dataset is the largest in this study and derived from a publicly available resource designed for genomic prediction models [64]. This dataset originally comprised 3,534 individuals with high-density genotypes and continuous phenotypes of five anonymized traits. The SNP data were anonymized by randomizing the map order. After cleaning some missing data, we finally obtained genomic marker data from 2,314 individuals, and each sample contains 52,843 SNPs with 0, 1, and 2 coding. The pedigree structure of this dataset is relative complex, spanning multiple generations of ancestral relationships. The number of individuals in the pedigree of the pig data is 6473 which means that there are considerably less individuals with the genomic markers. The elbow point of the eigenvalues in the scree plot were used to select the most relevant components which turned out to be 797 eigenvectors (Fig. 2c). These were then transformed into pedigree loadings, which were integrated into a joint dataset with genomic markers and pedigree loadings for evaluation similar to the two other species.

## Data Availability

The code for the GRVSNN models and the datasets are available at https://github.com/angelYHF/GRVSNNs The original data sets are available at: Mice data: https://cran.r-project.org/web/packages/BGLR/ Pig data: https://academic.oup.com/g3journal/article/2/4/429/6026060/ Loblolly pine data: https://academic.oup.com/genetics/article/190/4/1503/6064084/
